# Growth hormone signaling is necessary for lifespan extension by dietary methionine

**DOI:** 10.1111/acel.12269

**Published:** 2014-09-19

**Authors:** Holly M Brown-Borg, Sharlene G Rakoczy, Joseph A Wonderlich, Lalida Rojanathammanee, John J Kopchick, Vanessa Armstrong, Debbie Raasakka

**Affiliations:** 1 Department of Basic Sciences, University of North Dakota School of Medicine and Health SciencesGrand Forks, ND, 58203, USA; 2 Department of Biomedical Sciences Heritage College of Osteopathic Medicine, Edison Biotechnology Institute, Ohio UniversityAthens, OH, 45701, USA

**Keywords:** dwarf, lifespan, growth hormone, methionine, mice

## Abstract

Growth hormone significantly impacts lifespan in mammals. Mouse longevity is extended when growth hormone (GH) signaling is interrupted but markedly shortened with high-plasma hormone levels. Methionine metabolism is enhanced in growth hormone deficiency, for example, in the Ames dwarf, but suppressed in GH transgenic mice. Methionine intake affects also lifespan, and thus, GH mutant mice and respective wild-type littermates were fed 0.16%, 0.43%, or 1.3% methionine to evaluate the interaction between hormone status and methionine. All wild-type and GH transgenic mice lived longer when fed 0.16% methionine but not when fed higher levels. In contrast, animals without growth hormone signaling due to hormone deficiency or resistance did not respond to altered levels of methionine in terms of lifespan, body weight, or food consumption. Taken together, our results suggest that the presence of growth hormone is necessary to sense dietary methionine changes, thus strongly linking growth and lifespan to amino acid availability.

## Introduction

Determinants of lifespan in mammals include aspects of both genetics and the environment. The longevity impact of environmental manipulations in rodents has included studies that have changed temperature, activity opportunities (i.e. exercise), and dietary intake in terms of amount, composition, or availability (i.e. *ad libitum* every other day feeding). The proposed mechanisms underlying the longevity effects of dietary restriction include enhanced stress defenses and insulin sensitivity among others. Limiting specific nutrients such as methionine in the diet also extends lifespan without reductions in food consumption, even when introduced midlife (Orentreich *et al*., 1993; Miller *et al*., 2005; Sun *et al*., 2009). Combining genetic and environmental approaches in the study of aging and lifespan provides a greater understanding of the effects of their interactions and may be important in the translation to humans.

Ames dwarf mice live more than a year longer than their wild-type siblings due to a point mutation in a gene involved in anterior pituitary differentiation (*Prop-1*) resulting in mice of diminutive size and enhanced insulin sensitivity and stress resistance (Brown-Borg *et al*., 1996; Bartke & Brown-Borg, 2004). These beneficial physiological manifestations are primarily attributed to the deficiency in circulating growth hormone (GH) observed in these long-living mice (Brown-Borg, 2009). Reducing the dietary intake (via calorie restriction) in these mice further increases lifespan (Bartke *et al*., 2001). Ames mice exhibit atypical methionine metabolism that results in significant enhancement of methionine cycling (Uthus & Brown-Borg, 2003, 2006). In liver tissue, the flux of methionine to the transsulfuration pathway is three times higher in dwarf mice, while transmethylation is two times that observed in wild-type mice. In turn, glutathione levels are higher in several tissues of Ames mice and likely contribute to the overall observed increase in stress defense mechanisms. Moreover, we demonstrated that the administration of GH to GH-deficient Ames mice suppressed components of the methionine pathway (Brown-Borg *et al*., 2005).

GH action has a major impact on lifespan in rodents. In addition to the longevity established in GH-deficient rodents (Ames, Snell, Little, GHRH KO), mice generated to express dysfunctional GH receptors (GH receptor knockout; GHRKO) exhibit greater median and maximum lifespans as well (Coschigano *et al*., 2000; Flurkey *et al*., 2001; Sun *et al*., 2013). In stark contrast, mice that overexpress GH live about half as long (∼12 months) as their wild-type controls (Steger *et al*., 1993). Similar to Ames mice, the GHRKO and the GH transgenic animals display alterations in the methionine metabolic pathway (Brown-Borg *et al*., 2009; H. M. Brown-Borg, unpublished data). Thus, we hypothesized that lifespan would be differentially affected by dietary methionine levels in these GH mutant strains. Furthermore, there were very few reports using methionine supplementation in the context of aging and none of which provided lifespan data in rodents (Gomez *et al*., 2009). Therefore, these studies were conducted to evaluate the relationships between methionine restriction, methionine supplementation, and GH status on longevity.

## Results

Beginning at 8 weeks of age, three levels of methionine were provided to mice to represent a severe restriction (0.16%), approximately 50% reduction (0.43%) compared with standard rodent diets and more achievable in human diets, and an enriched level (1.3%) that remained below toxicity levels. The data were not compared directly across mutants due to background strain variation.

### Lifespans of Ames dwarf, GHRKO, and GH transgenic mice

Median lifespan for Ames mice subjected to 0.16% MET was 859 days for dwarf (95% CI 686–974 days) and was 787 days for wild-type mice (95% CI 726–877 days; Fig.1A). Median lifespan was not significantly different between dwarf and wild-type mice. The 90th percentile survival represents a test for maximal longevity where the proportion of mice alive in each group is compared with the mice alive in the last 10% of the pooled population. These values provide a means of late-life comparison among diets within genotype, separating out potential early- and late-life effects. There was no difference in maximal longevity for Ames mice (1162 days) compared with wild-type (1087 days) on the 0.16% MET diet. The longest living wild-type mouse reached 1180 days of age, while the longest living dwarf mouse lived until 1417 days of age. At the time of publication, one dwarf mouse remained alive (1417 days).

**Figure 1:**
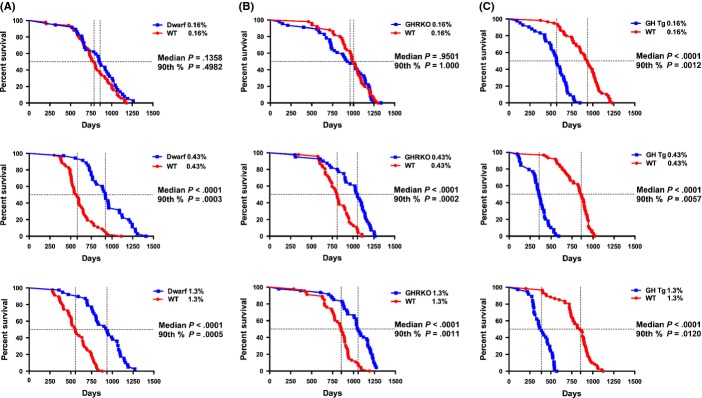
Survival curves for mutant mice on three levels of dietary methionine. (A) Ames dwarf and wild-type mice. (B) GHRKO and wild-type mice. (C) GH transgenic and wild-type mice. Each panel represents the response of the mice to 0.16%, 0.43%, or 1.3% methionine, and symbols represent individual mice. Vertical dashed lines represent median lifespan for each genotype. Median and 90th percentile statistics for genotype are shown on graph. At the time of publication, 1/37 dwarf (1417 days) and 0/50 wild-type mice were alive in the 0.16% group, 0/35 dwarf and 0/46 wild-type mice were in the 0.43% group, and 1/39 dwarf (1359 days) and 0/47 wild-type mice were left in the 1.3% MET group. At the time of publication, 0/45 GHRKO and 0/50 wild-type mice were alive (0.16%), 0/40 GHRKO and 0/44 wild-type mice were in the 0.43% group, and 2/47 GHRKO (1289, 1289 days) and 0/46 wild-type mice were left in the 1.3% MET group. 0/41 GH Tg and 0/60 wild-type mice on the 0.16% MET were alive, 0/39 GH Tg and 0/60 wild-type mice on 0.43% MET were alive, and 0/37 GH Tg and 0/60 wild-type mice were left on 1.3% MET.

Median lifespan for dwarf mice consuming the 0.43% MET diet was 920 days (95% CI 799–1089 days). Wild-type mice exhibited median lifespans of 581 days of age (95% CI 512–647 days). Dwarf mice retained significant median lifespan extension over that of wild-type mice on this diet (58%). Maximal lifespans for dwarf mice were also greater (45%) than those of wild-type mice consuming the 0.43% methionine diet (1284 vs. 888 days). The longest living wild-type mouse in this group reached 1113 days of age, while the oldest dwarf mouse lived up to 1410 days.

On the 1.3% MET-enriched diet, Ames dwarf mice outlived wild-type mice by 69% with median lifespans for Ames mice at 937 days (95% CI 799–1068 days) and 556 days (95% CI 505–649 days) for wild-type mice. The dwarf mice also exhibited a 48% extension in maximal lifespan compared with wild-type mice on this diet (1184 vs. 799 days, dwarf and wild-type mice, respectively). In the wild-type group, the longest lived mouse was 879 days of age, while in the dwarf group, a mouse lived up to 1359 days. At the time of publication, there was one dwarf mouse alive (1359 days).

The GHRKO mice are also considered a long-living GH mutant and share several characteristics with the Ames mice. Wild-type and mutant mice responded similarly to the 0.16% MET diets with no difference in median lifespan (Fig.1B). Median lifespan for the GHRKO mice was 946 days (95% CI 743–1126 days), while the wild-type mice lived 1004 days (95% CI 924–1061 days). GHRKO mice (1210 days) surviving past the 90th percentile did not differ from the wild-type mice (1231 days) when fed a diet containing 0.16% methionine. The last GHRKO mouse reached 1335 days, and the last wild-type mouse in this group lived until 1298 days.

Median lifespan for GHRKO mice on 0.43% MET was 30% longer at 1053 days (95% CI 915–1127 days), while wild-type mice lived 811 days (95% CI 749–903 days). Maximal longevity for the GHRKO (1216 days) also exceeded that of wild-type mice by 15% (1053 days). The longest living wild-type and GHRKO mice on the 0.43% MET diet lived 1108 and 1262 days, respectively.

Median lifespan remained greater in GHRKO when animals were fed a diet enriched for methionine (1.3%). GHRKO mice lived 1055 days (95% CI 1010–1170 days) and wild-type mice lived 854 days (95% CI 772–906 days), representing a 24% difference. The maximal longevity for the GHRKO mice (1254 days) also remained 19% greater than wild-type mice (1056 days) on the same diet. The longest living wild-type mouse on this diet lived 1189 days and the longest lived GHRKO mouse at the time of publication was 1289 days of age. There were two GHRKO mice alive at the time of publication (1289, 1289 days).

The GH transgenic mice survive about half as long as wild-type controls on normal rodent chow (12 months). When methionine is severely restricted (0.16%), GH transgenic mice maintained shortened (39%) median lifespans compared with wild-type mice but lived 6 months longer than previously reported (Fig.1C). Median lifespan for the GH transgenics was 569 days (95% CI 505–650 days), and for wild-type mice, it was 935 days (95% CI 851–1010 days) on the restricted MET diet. Maximal longevity for the GH transgenic mice was 761 days, significantly shorter (36%) than wild-type mice (1183 days). The longest living GH transgenic mouse lived 847 days, while the longest living wild-type mouse on this diet lived 1231 days.

On a less restrictive MET diet (0.43%), median lifespan in GH transgenics vs. wild-type mice differed significantly. Median lifespan for GH transgenic mice was 357 days (95% CI 315–412 days) and 860 days (95% CI 767–899 days) for wild-type mice, demonstrating a 58% difference. Maximal longevity differed (45%) between genotypes on this diet (GH Tg 532 days vs. wild-type 965 days). In the GH transgenic group, the longest lived mouse was 590 days of age, while the longest lived wild-type counterpart was 1022 days of age.

Increasing the methionine content to 1.3% also affected median and maximal lifespans. GH transgenic mice consuming 1.3% MET lived 387 days (95% CI 330–462 days), and wild-type mice lived 855 days (95% CI 758–897 days), representing a 55% difference between genotypes. Maximal longevity for the wild-type mice (1017 days) vs. the GH transgenic mice (539 days) also differed by 47%. The longest lived GH transgenic on the MET-enriched diet was 569 days of age, and for the wild-type mice, the oldest mouse was 1122 days of age.

### Lifespan differences between diets and interactions

Examining the differences between diets within genotypes provides insight as to how each genotype responds to the varied levels of methionine (Fig.2). One key finding was that within genotype, the median and maximal lifespans of Ames dwarf and GHRKO mice consuming all three levels of methionine were not different from one another. The median lifespans of each of the three lines of wild-type mice on the lowest level of methionine (0.16%) were markedly extended compared with animals on the higher MET diets. Maximal longevity was also extended between these groups. Similar to the wild-type mice in each line, GH transgenic mice benefitted significantly from the lowest level of dietary methionine in terms of median and maximal lifespan living >40% longer than transgenic mice exposed to higher levels of methionine.

**Figure 2:**
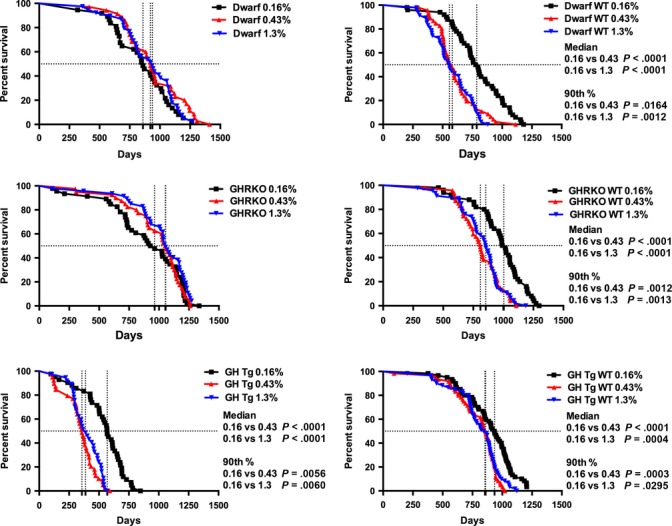
Survival curves for all mice compared across diets within genotype. Each panel represents the genotype response to each level of dietary methionine. Symbols represent individual mice, and vertical lines indicate median lifespan. Median and 90th percentile lifespan differences between diets are indicated.

Analyses of the influence of MET concentration on longevity in the Ames dwarf mouse and its wild-type counterpart revealed a significant influence of genotype and dietary MET on median lifespan (Fig.3). The dwarf mouse lived longer than the wild-type (*F*_1,248_ = 82.25; all *P*-values on graph), and the severely restricted diet, 0.16% MET, was associated with greater longevity than the diets of 0.43% or 1.3% MET (*F*_2,248_ = 3.648). However, these main effects should be interpreted within the context of a significant genotype x diet interaction (*F*_2,248_ = 12.12). The Ames dwarf mouse displayed greater longevity than the wild-type on both the 0.43% and 1.3% diets, but exhibited no difference between genotype and lifespan in the 0.16% condition. The influence of dietary MET level on lifespan in the GHRKO and their wild-type controls indicated significant effects of genotype (*F*_1,277_ = 12.57), but no significant influence of diet (*F*_2,277_ = 0.6261). Again, the main effect of genotype is impacted by a significant genotype × diet interaction (*F*_2,277_ = 10.90). The GHRKO displayed greater longevity in the 0.43% and 1.3% diets than the wild-type, but showed no difference in lifespan in the 0.16% condition. The GH transgenic line demonstrated a significant effect of genotype and diet on longevity. Specifically, the wild-type mouse lived longer than the GH transgenic (*F*_1,299_ = 383.2), and all mice, in either condition, lived longer on the 0.16% than the mice on the 0.43% or 1.3% methionine conditions (*F*_2,292_ = 20.31).

**Figure 3:**
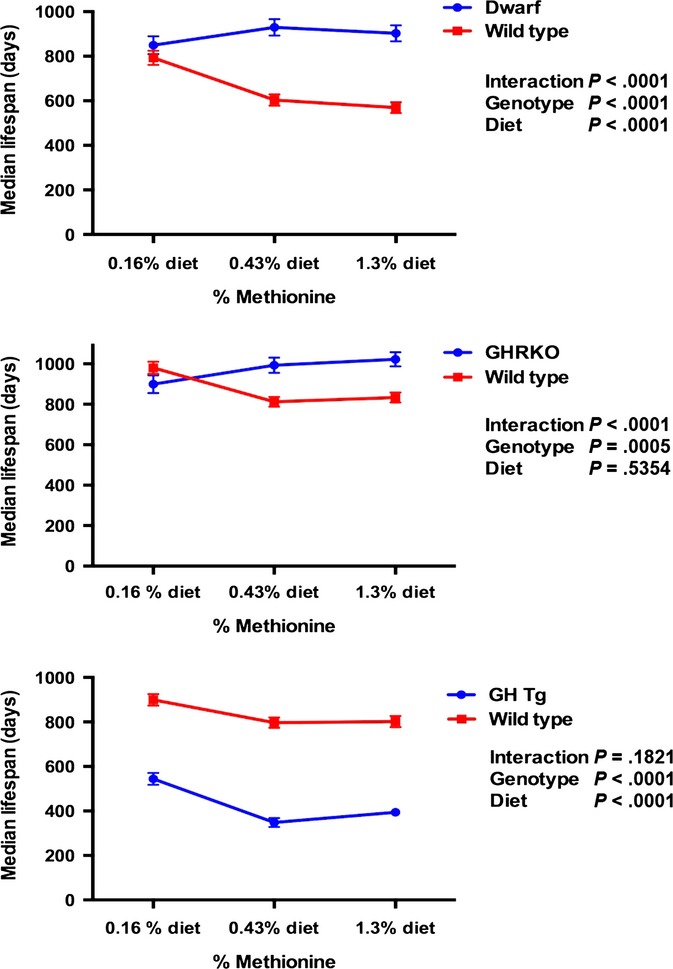
The interactions between genotype and dietary methionine on lifespan for each GH mutant mouse line. The results of two-way ANOVAs are shown on each graph.

### Body weights and food consumption

Body weights were recorded throughout the study beginning at 8 weeks of age (Fig.4). Overall, mean body weights of dwarf mice were maintained and mostly unaffected by different levels of dietary MET, similar to the limited effects of this diet on lifespan. In the last quartile (75% Q), body weights were different among the animals on the lowest level of MET compared with the others (0.43% *P* < 0.01, 1.3% *P* < 0.001). Wild-type mice on the 0.16% MET maintained their body weight for nearly their entire lifespan, while those on the 0.43% and 1.3% shared similar patterns of weight gain until 15 months of age (25% Q, 50% Q, and 75% Q quartiles; 0.43% *P* < 0.0001, 1.3% *P* < 0.0001). The patterns of weight gain and loss in GHRKO mice were nearly identical between diets (Fig.4) with no differences in quartile lifespan body weights detected between diets. The wild-type mice in this line maintained their body weights when consuming the 0.16% MET diet, while those animals fed the higher MET levels gained weight until 18 months of age (25% Q, 50% Q, and 75% Q; 0.43% *P* < 0.0001, 1.3% *P* < 0.0001). Growth hormone transgenic mice are much heavier at 8 weeks of age compared with the other genotypes in this study. Transgenic mice consuming the 0.43% and 1.3% MET diets rapidly gained weight until 9 months of age, while those on the restricted MET diet gained weight more slowly and started losing weight after 22 months of age (Fig.4). The patterns of body weight maintenance and quartile lifespan differences were similar to those of wild-type animals in the Ames dwarf and GHRKO lines although compressed in time. Animals on the 0.16% diet differed at each lifespan quartile (25, 50, and 75%) when compared with the other two diets (0.43% *P* < 0.0001, 1.3% *P* < 0.0001). Body weights of wild-type mice in the transgenic line followed similar patterns (and quartile lifespan differences) with mice on the 0.16% differing from those on higher levels of MET (0.43% *P* < 0.0001, 1.3% *P* < 0.0001).

**Figure 4:**
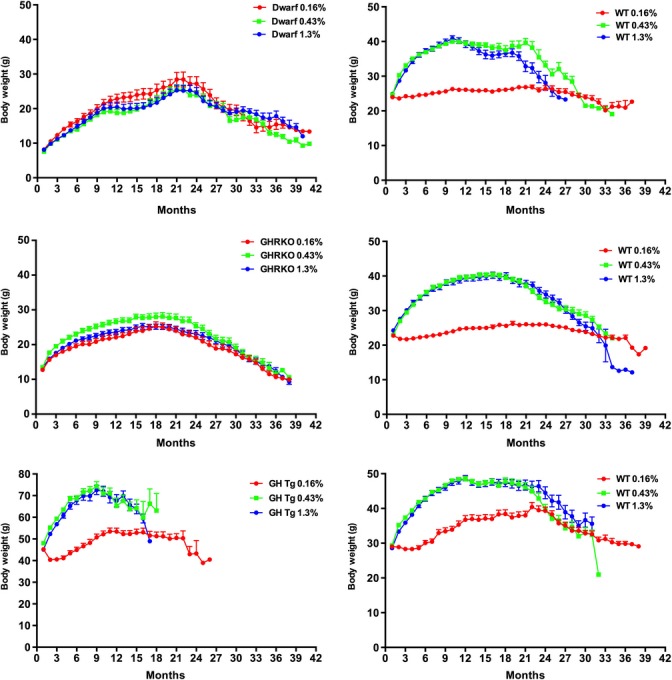
Body weights of all genotypes on different levels of dietary methionine from 2 months of age until a month within death. Symbols represent mean body weight/month ± SEM for individual genotypes on each diet. Body weights at each quartile of lifespan are compared statistically and noted in the text.

Food consumption was measured in each genotype for 6 weeks beginning at 8 weeks of age as previous studies suggested that palatability issues may play a role in altered MET diets (Fig.5). Within genotype, Ames dwarf and GHRKO mice ate similar amounts of diet, regardless of the MET content. Previous reports indicate that Ames mice eat more per gram of body mass compared with wild-type mice and thus are not voluntarily calorie restricted (Mattison *et al*., 2000). Wild-type mice in all three lines consumed more diet with 0.16% MET when compared with the diets containing higher levels of MET. GH transgenic mice on the 0.16% diet ate 70–80 g of food per week. The amount of food consumed by wild-type mice in this line varied by week and diet (significant interaction *P* = 0.0003).

**Figure 5:**
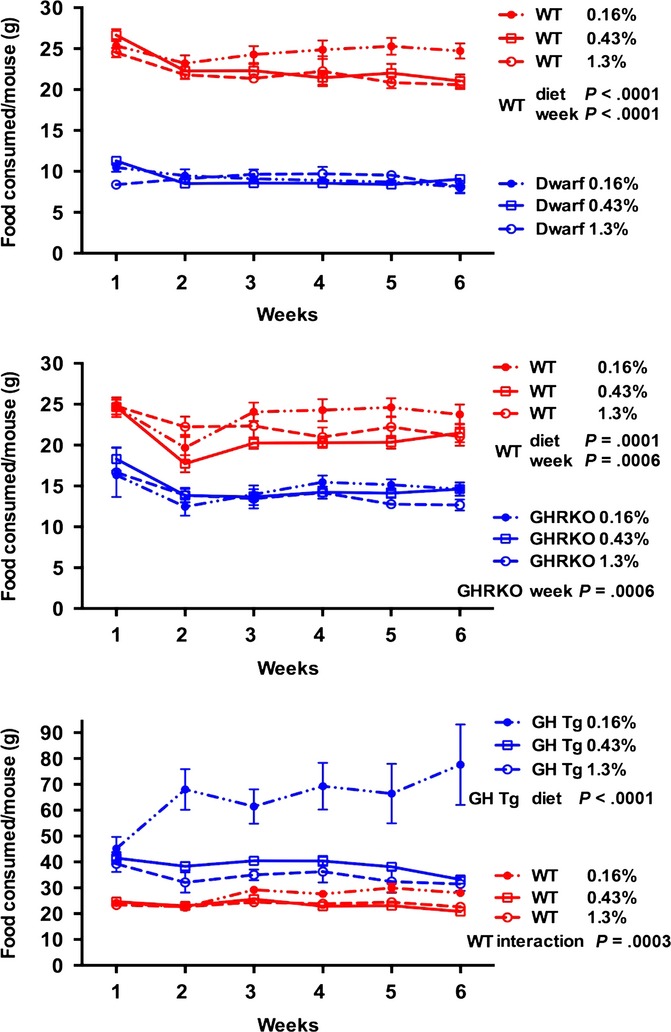
Food consumption per mouse (grams) for each GH mutant and wild-type control on three levels of dietary methionine during the first 6 weeks of study. Each symbol represents the mean ± SEM of all mice in each group.

### Pathology

Gross necropsy results indicated that higher MET levels resulted in greater numbers of liver tumors in all wild-type and GH mutant mice (Table1). When all tumors for each genotype on diet were calculated, the tumor burden was observed to be significantly affected by diet with decreased tumor burden in animals on the 0.16% diet (*P* < 0.0001; data not shown). A consideration that the age at death was much greater in the Ames and GHRKO groups explains the lack of a significant genotype effect on the presence of liver tumors compared with wild-type mice and confirms earlier reports (Ikeno *et al*., 2003, 2009). Nearly 50% of the GH transgenic mice on the highest level of MET exhibited markedly enlarged bladders at death with the percentage decreasing with decreasing MET (interaction *P* = 0.0031).

**Table 1:** Pathology at death of (A) Ames dwarf; (B) GHRKO; and (C) GH transgenic and respective wild-type mice fed 0.16%, 0.43%, and 1.3% methionine beginning at 8 weeks of age

Pathology	0.16% dwarf (*n* = 32)	0.16% wild-type (*n* = 44)	0.43% dwarf (*n* = 28)	0.43% wild-type (*n* = 42)	1.3% Dwarf (*n* = 33)	1.3% wild-type (*n* = 44)	*P*-value diet	*P*-value genotype
(A)								
Liver tumors	13 (41%)	17 (39%)	13 (46%)	20 (48%)	19 (58%)	24 (55%)	0.0100	0.8603
Lung tumors	7 (22%)	12 (27%)	9 (32%)	21 (50%)	8 (24%)	16 (36%)	0.1065	0.0640
Kidney tumors	0	5 (11%)	1 (4%)	7 (17%)	3 (9%)	5 (12%)	0.4889	0.0711
Enlarged bladder	0	8 (18%)	1 (4%)	2 (5%)	0	7 (16%)	0.4808	0.0009
Pathology	0.16% GHRKO (*n* = 34)	0.16% wild-type (*n* = 39)	0.43% GHRKO (*n* = 29)	0.43% wild-type (*n* = 39)	1.3% GHRKO (*n* = 34)	1.3% wild-type (*n* = 40)	*P*-value diet	*P*-value genotype
(B)								
Liver tumors	13 (38%)	15 (38%)	19 (66%)	28 (72%)	20 (59%)	24 (60%)	<0.0001	0.3119
Kidney tumors	1 (3%)	3 (8%)	0	2 (5%)	1 (3%)	6 (15%)	0.2161	0.0247
Enlarged bladder	0	4 (10%)	0	2 (5%)	0	4 (10%)	0.7199	0.0035
Kyphosis	13 (38%)	22 (56%)	6 (21%)	14 (36%)	13 (38%)	19 (48%)	0.0504	0.0490
Pathology	0.16% GH Tg (*n* = 37)	0.16% wild-type (*n* = 55)	0.43% GH Tg (*n* = 33)	0.43% wild-type (*n* = 57)	1.3% GH Tg (*n* = 33)	1.3% wild-type (*n* = 58)	*P*-value diet	*P*-value genotype
(C)								
Liver tumors	21 (57%)	11 (20%)	21 (64%)	29 (51%)	23 (70%)	37 (64%)	<0.0001	0.0093
Mammary tumors	1 (3%)	1 (2%)	2 (6%)	4 (7%)	1 (3%)	0	0.0431	0.3689
Kidney tumors	2 (5%)	9 (16%)	8 (24%)	18 (32%)	4 (12%)	20 (35%)	0.0050	0.0078
Enlarged bladder	4 (11%)	19 (35%)	14 (37%)	17 (30%)	16 (49%)	13 (22%)	0.0486*	0.6302*

* Interaction *P* = 0.0031.

## Discussion

Longevity is significantly influenced by the endocrine system and specifically by components of the somatotropic axis. In this study, the interaction between GH status and methionine was of interest as the lack of GH-induced signaling and low dietary methionine, independently, is known to extend lifespan. In contrast, high-plasma GH and high dietary methionine shorten lifespan and induce severe hepatitis, respectively (Steger *et al*., 1993; Yamada *et al*., 2012). Others have reported that GH decreases key enzymes in the MET pathway (Aida *et al*., 1997; Oscarsson *et al*., 2001). Taken together, the evidence suggests that a significant relationship between plasma GH and MET metabolism exists.

Methionine is an essential amino acid in mammals, and restricting MET in the diets of rodents extends lifespan (Orentreich *et al*., 1993; Miller *et al*., 2005; Sun *et al*., 2009). However, excess dietary methionine decreases food intake and growth and induces significant liver pathology (Yalcinkaya *et al*., 2009). Methionine is necessary for the synthesis of S-adenosylmethionine (SAM), the major biological methylating agent, and serves as the source of cysteine residues for glutathione biosynthesis (Fukagawa, 2006). Deficiencies in MET or other factors that contribute to folate metabolism are associated with developmental abnormalities and several diseases (Mato *et al*., 2008).

The Ames dwarf and GHRKO mice are long-living GH mutant strains that exhibit atypical methionine metabolism (Uthus & Brown-Borg, 2003, 2006; Brown-Borg *et al*., 2009) and thus may respond differently to altered levels of dietary MET. Ames dwarf and GHRKO mice lived a similar length of time as their wild-type controls when fed the 0.16% MET. Importantly, this finding reflects both a lack of response to low MET by the GH signaling-deficient mice and a significant extension of lifespan by their respective wild-type mice. On higher levels of MET, both the GHRKO and Ames mice outlived (median) their wild-type counterparts by 7–8 and 11–12 months, respectively. Maximal longevity did not differ between GHRKO or Ames mice, regardless of diet. Collectively, the data suggest that without circulating GH or GH action, these animals do not discriminate between the differing levels of dietary MET as do animals with circulating plasma GH.

Ames dwarf mice utilize MET at higher rates than wild-type controls (Uthus & Brown-Borg, 2006). MET metabolism is also enhanced in the GHRKO and Snell dwarf mice (Brown-Borg *et al*., 2009; Vitvitsky *et al*., 2013). The increased rate of MET metabolism (due to the lack of GH) may override the normal regulatory mechanisms that modulate enzyme activity to maintain substrates within a limited range (Finkelstein, 2000; Martinov *et al*., 2000). Our preliminary data suggest that the Ames mice maintain higher levels of the key enzymes in this pathway, regardless of diet (H. M. Brown-Borg, unpublished data). Conversely, many of the components of the MET pathway are suppressed in the short-living GH transgenic mice (H. M. Brown-Borg, unpublished data). These animals with high circulating GH levels responded favorably to the low dietary MET living 6 months longer than that reported (Steger *et al*., 1993). They did not live as long as the wild-type mice in this line as these animals also exhibited enhanced longevity on MET restriction. Together with the evidence in the GH signaling mutants, we find that the activity of the MET pathway is highly correlated with GH levels and likely impacts the ability of the system to sense changes in dietary methionine.

Animals exhibiting GH action (wild-types and GH transgenics) responded positively to dietary MET restriction in terms of median and maximal lifespan. No differences within genotype were detected in diets containing higher levels of MET. Neither Ames dwarf nor GHRKO mice, animals lacking GH signaling, responded to methionine restriction with lifespan extension (median or maximal). We found that the effect of genotype on longevity was moderated by the amount of dietary MET in both Ames and GHRKO mice. Under conditions of 0.43% and 1.3% MET, the dwarf and GHRKO exhibited longer life. However, when dietary MET was limited to 0.16%, the significant difference between the GH signaling mutants and their wild-type controls no longer existed.

Animals lacking GH action did not distinguish the dietary MET differences in terms of body weight or food consumption. However, mice with normal or high-plasma GH were able to discriminate between low MET and higher levels of dietary MET by increasing food consumption but not gaining significant body weight when consuming 0.16% MET. Increases in total energy expenditure may explain the lack of weight gain in concert with increased food consumption in MET-restricted animals (Malloy *et al*., 2006; Hasek *et al*., 2010). In agreement, Ames dwarf and GHRKO mice (on standard laboratory chow) exhibit decreased respiratory quotients and increased VO2 compared with wild-type controls (Bartke & Westbrook, 2012).

Ames and GHRKO mice did not alter consumption based on dietary methionine content. GH transgenic mice grew at a much faster rate and ate twice as much of the MET-deficient diet in an effort to maintain their accelerated growth. This information supports the need for GH in the perception of nutrient intake, specifically methionine. The selective sensitivity of the GH system to methionine may ensure that the drive to grow is modulated not by general amino acid availability, but by the amino acid least available in the nutrient supply (Richardson & Hatfield, 1978). A decrease in methionine would critically affect protein synthesis capacity, a physiological function that GH actively stimulates. Without GH, the drive for protein synthesis is decreased, and thus, low dietary methionine may not exert the same downstream effects as occurs when adequate or high GH is present. It is possible that MET restriction acts similarly. The absence of GH or deficiency in methionine may each, independently, shift metabolic resources from an emphasis on growth and proliferation to cellular defense and protection. A limitation of our study was the manual measurement of food intake, which is somewhat less accurate than an automated one. We cannot exclude that these measures could slightly affect their behavior and food intake assessment. In addition, when an estimate of the total amount of MET consumed over median lifespan for each genotype and diet was calculated, only the GH Tg mice fed 0.16% MET ate greater amounts of diet when compared with their wild-type counterparts (data not shown). These estimated MET consumption values did not correlate with lifespan.

We showed that with alternate day feeding, methionine recycling was enhanced in wild-type and dwarf mice, while transsulfuration was significantly decreased similar to that reported in mice fed low MET and in rats fed high fat (Finkelstein *et al*., 1988; Tang *et al*., 2010; Brown-Borg & Rakoczy, 2013; Dahlhoff *et al*., 2013), effectively decreasing transsulfuration to conserve MET. A potential link between these observations involves the peroxisome proliferator-activated receptor (PPAR) gene family. Many peroxisome proliferator-regulated genes are under the control of GH, and PPARα is constitutively upregulated in dwarf mice (Stauber *et al*., 2005). PPARα is a regulator of hepatic amino acid metabolism and has been shown to suppress the transsulfuration pathway (Rakhshanderoo *et al*., 2010). In the Ames mice, remethylation is suppressed in part by GH, and thus, the carbon backbone of methionine is forced through the transsulfuration pathway maintaining increased activity of these enzymes (Brown-Borg *et al*., 2005). Therefore, in the absence of GH, the methionine cycle is greatly perturbed both in terms of normal allosteric and feedback functions and in the ability of the system to sense dietary levels of this amino acid. In support of a role of the GH and dietary MET sensing, we found that IGF1 levels decline in wild-type and GH transgenic mice fed diets restricted in MET (Fig.6; Miller *et al*., 2005; Malloy *et al*., 2006). A target of IGF1, mammalian target of rapamycin (mTOR), acts as an intracellular sensor of nutrient status and as a regulator of protein synthesis. mTOR is downregulated in Ames and GHRKO mice (Sharp & Bartke, 2005; Wang & Miller, 2012). However, in MET restriction, mTOR activity is not decreased, suggesting that additional mechanisms of nutrient sensing may be in play (Sun *et al*., 2013).

**Figure 6:**
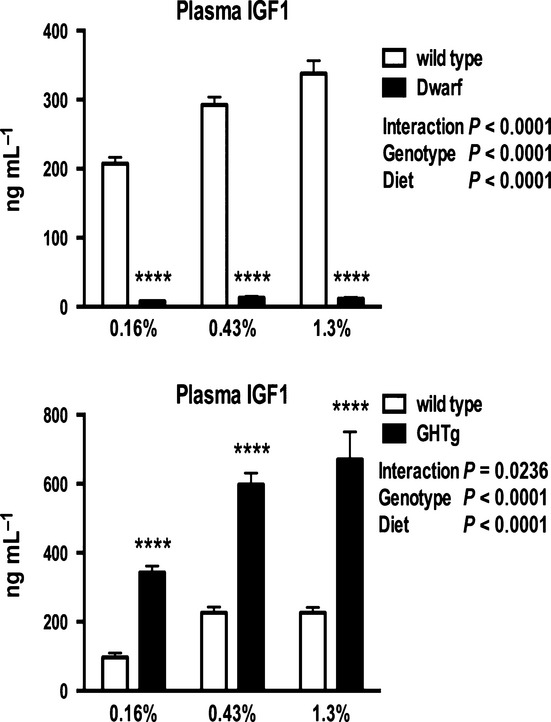
Plasma insulin-like growth factor 1 levels in Ames dwarf, GH transgenic mice, and respective wild-type mice on 0.16%, 0.43%, or 1.3% methionine for 8 weeks. ****P < 0.0001.

A lifetime of excess plasma GH leads to significant disease burden and premature death (von Waldthausen *et al*., 2008). Dietary MET levels significantly impacted the presence of tumors as well as enlarged bladders in GH transgenic mice. With regard to animals with circulating GH, MET restriction appears to delay the onset of disease, similar to previous reports (Miller *et al*., 2005).

## Conclusion

Longevity is significantly influenced by the somatotropic axis. Here, we show that active GH signaling is necessary for mice to respond to changes in dietary methionine in terms of lifespan, body weight, and food consumption. The survival curves of mice with normal or excess plasma GH levels appeared similar. In contrast, the lifespans of Ames dwarf and GHRKO mice indicate that without GH signaling, the system is unable to detect or sense changes in dietary methionine. Thus, the underlying genotype effects that result in a lack of GH signaling are not apparent when animals consume low MET diets. In cases of either GH or MET deficiency, metabolic reprogramming occurs possibly shifting resources away from growth toward more protective mechanisms, resulting in lifespan extension.

## Experimental procedures

Ames dwarf mice were derived from a closed colony with a heterogeneous background (over 25 years) at the University of North Dakota. Dwarf mice were generated by mating either homozygous (df/df) or heterozygous (df/+) dwarf males with carrier females (df/+). Male and female dwarf mice were included to increase cohort numbers as the initial analyses to control for gender showed no differences in either gender on any of the diets. Thus, further analyses were carried out with pooled data of both genders. Male wild-type and GHRKO mice (generated by Zhou *et al*., 1997; kindly provided by Andrzej Bartke) were developed by crossing 129Ola/BALB/c normal (GHR+/−) animals with mice derived from crosses of C57BL/6J and C3H/J strains and maintained as a closed colony (Panici *et al*., 2009). The male GH transgenic mice (kindly provided by Andrzej Bartke) were derived from a single male founder (strain B6SJL) produced by microinjection of the phosphoenolpyruvate carboxykinase (PEPCK) promoter region/bGH hybrid gene into the male pronucleus of single-cell embryos. The production and initial characterization (transgenic males crossed to C57Bl/6J × C3H/J F1 females) were previously described (McGrane *et al*., 1988). Forty to sixty mice of each genotype were utilized in the lifespan studies. All mice were bred and maintained at the University of North Dakota Center for Biomedical Research under controlled conditions of photoperiod (12:12 h light/dark cycle) and temperature (22 ± 1 °C) and *ad libitum* access to food (birth to 8 weeks of age; Teklad #8640) and water. Animal procedures were reviewed and approved by the UND Institutional Animal Care and Use Committee. Spontaneous (natural) death was used as the end point for survival data. Animals were checked daily and euthanized when considered to be within 48 h of natural death using the following criteria: (i) not able to eat or drink; (ii) bleeding from a tumor or other open sore condition; (iii) failure to move when prodded or unable to right themselves; or (iv) extreme distress due to inability to empty bladder. The presence or absence of gross pathology was determined in each animal.

Animals were started on the diets at 8 weeks of age. Three levels of methionine (0.16%, 0.43%, 1.3% MET) were incorporated into amino acid-defined diets manufactured by Harlan Teklad based on the AIN-93 diet (0.16%—TD.10230; 0.43%—TD.10231; 1.3%—TD.10232). Glutamic acid was adjusted to keep the diets isonitrogenous (suggested by Harlan Laboratories, Madison, WI, USA). l-methionine was the only source of sulfur amino acids in these diets. The 0.43% MET diet represented approximately 50% of the MET found in many rodent chows based on the AIN-76 diet series and a level of methionine that might be more achievable in human diets. A 1.3% methionine level was used to represent an enriched diet yet supplementing below the reported level of toxicity (Fukagawa, 2006; Yamada *et al*., 2012).

Body weights were recorded monthly until death. Food intake per cage was monitored over the first 6 weeks on the diets. The weight of the uneaten food (in hopper and on cage floor) in each cage was determined three times per week for 6 weeks and subtracted from the weight of the food originally placed in the hopper. Plasma IGF-1 levels were determined following 8 weeks on diets in separate groups of animals that were not included in the lifespan studies (Quantikine Mouse/Rat IGF-1 ELISA; R&D Systems, Minneapolis, MN, USA).

### Statistics

One-way or two-way ANOVAs were utilized to determine the effects of genotype and diet on body weight, food consumption, and incidence of pathology using Prism 6.0 (GraphPad). Within genotype, the body weights at each quartile (Q) of lifespan were compared across diets. Median lifespan was determined using Kaplan–Meier survival curves with log-rank (Mantel–Cox) tests. 90th percentile survival representing maximal lifespan was calculated and compared using Fisher's exact tests (Wang *et al*., 2004). These values provide a means of late-life comparison among diets within genotype, separating out the potential early- and midlife effects.
